# The role of autologous stem cell transplantation (ASCT) in aggressive B-cell lymphomas: real-world data from a retrospective single-center analysis

**DOI:** 10.1007/s00277-021-04650-5

**Published:** 2021-09-03

**Authors:** Ramona Wullenkord, Philipp Berning, Anna-Lena Niemann, Klaus Wethmar, Sarah Bergmann, Mathias Lutz, Christoph Schliemann, Rolf Mesters, Torsten Keßler, Norbert Schmitz, Wolfgang E. Berdel, Georg Lenz, Matthias Stelljes

**Affiliations:** Department of Medicine A, Hematology and Oncology, University Hospital Münster, Albert-Schweitzer-Campus 1, 48149 Münster, Germany

**Keywords:** Autologous stem cell transplantation, Aggressive B-cell lymphoma, DLBCL, HL, MCL, PCNSL, Prognostic factors

## Abstract

Patients with high-risk or relapsed aggressive B-cell lymphomas are characterized by poor prognosis. High-dose chemotherapy followed by autologous stem cell transplantation (ASCT) can induce durable remissions in these patients and is potentially curative. Two hundred forty-seven patients with aggressive B-cell lymphomas treated with high-dose chemotherapy and ASCT, either as consolidation after first-line therapy or after salvage therapy for relapsed disease, between 2002 and 2019 at the University Hospital Muenster, were analyzed. The median follow-up of surviving patients was 36 months (range 0–163). Progression-free survival (PFS) and overall survival (OS) after 3 years was 63% and 68%, respectively. After ASCT, 28% of all patients experienced a relapse. The cumulative incidence of non-relapse mortality at day 100 after ASCT was 4%. Multivariate analysis identified remission status at ASCT, age at ASCT, and the numbers of infused CD34^+^ cells as independent prognostic factors for both PFS and OS. Patients with mantle cell lymphoma (MCL) or primary CNS lymphoma (PCNSL) treated with ASCT in first-line had a superior OS and PFS when compared to patients treated with ASCT in relapsed disease. For patients with diffuse large B-cell lymphoma (DLBCL) and Hodgkin lymphoma (HL), early relapse (< 12 months) after first-line therapy showed a trend towards an inferior PFS and OS. Deaths after ASCT were predominantly caused by lymphoma relapse and/or progression (64%) or due to infections (23%). In conclusion, high-dose chemotherapy followed by ASCT in the era of novel targeted agents remains a feasible and effective approach for patients with high-risk or relapsed aggressive B-cell lymphomas. Remission status and age at ASCT, and the number of infused stem cells were of prognostic relevance.

## Introduction

Frontline immunochemotherapies induce complete remissions in the majority of patients with aggressive B-cell lymphomas. However, approximately 20–30% of patients with non-Hodgkin lymphomas and 15% with Hodgkin lymphomas (HL) eventually fail to achieve a complete remission or experience relapse after first-line therapy [1–6]. More intense therapies, e.g., the approach tested in the MegaCHOEP study evaluating high-dose (HD) chemotherapy as part of the first-line treatment for young patients with high-risk DLBCL, failed to show survival benefit when compared to conventional R-CHOP-like therapies [7].

Patients with relapsed or refractory aggressive lymphoma have a dismal prognosis, but effective salvage regimens followed by HD chemotherapy and autologous stem cell transplantation (ASCT) still offer curative options for these patients [8–10]. In patients with relapsed or refractory diffuse large B-cell lymphoma (DLBCL), event-free survival rates at 4 years after ASCT range between 40 to 50% [8, 11]. Similarly, in relapsed HL, salvage treatment including HD chemotherapy and ASCT can induce long-term progression-free survival (PFS) rates of 50 to 60% at 5 years after ASCT [12–14].

For primary CNS lymphoma (PCNSL) and mantle cell lymphoma (MCL), consolidation with HD chemotherapy followed by ASCT in the first-line treatment still represents the standard of care in many Western countries [15–18]. In MCL, patients receiving HD chemotherapy consolidation showed improved PFS (54% vs. 25%) and OS (83% vs. 77%) at 3 years after ASCT compared to patients treated with interferon alpha-based maintenance https://pubmed.ncbi.nlm.nih.gov/15591112/. For PCNSL, a similar role of ASCT has been demonstrated in recent trials [15, 19].

Infections and mucositis represent the most relevant adverse events after ASCT, while other toxicities including impairment of renal and liver function are less prevalent [20, 21]. Non-relapse mortality varies between 1 to 4% at day 100 after ASCT [21, 22]. Death after ASCT is mostly related to relapse or progression of lymphoma, followed by infections, organ failure, and secondary malignancies [23].

To date, there is only limited data on risk factors predicting post-ASCT outcome. Several prospective studies identified early relapse, generalized or systemic disease, and persistent disease at ASCT to be associated with inferior post-ASCT outcome [2, 8, 14]. Here, we sought to investigate the prognostic impact of these factors and aimed to identify additional prognostic features in a real-world setting. In this retrospective analysis, we analyzed the clinical course and outcome of 247 patients with aggressive B-cell lymphomas, including MCL, DLBCL, PCNSL, and HL, who received HD chemotherapy followed by ASCT at our center between 2002 and 2019.

## Methods

### Patients

For this retrospective analysis, we identified patients diagnosed with DLBCL, MCL, HL, and PCNSL who received HD chemotherapy and ASCT at the Department of Hematology and Oncology of the University Hospital Muenster. All patients received autologous peripheral blood stem cells and rituximab-based first-line treatment. Patients with secondary DLBCL after transformation of indolent lymphoma were included. Patients with indolent lymphoma as indication for ASCT, Richter’s transformation of CLL/SLL, concurrent other neoplastic disease without remission, and patients not responding to salvage treatment prior to ASCT were excluded. Histological diagnoses were confirmed based on available pathological reports. Detailed information on molecular marker profiles as well as material for additional testing was not available. The choice of induction or salvage treatment was at the discretion of the treating physician. Computed tomography (CT) or positron-emission-tomography combined with CT (PET-CT) was used to assess tumor response. However, PET-CT data for response assessment was limited to a small fraction of the reported cases. For PCNSL, CT- or MRI-based imaging techniques were accepted for disease evaluation. Response assessment was retrospectively determined according to the Lugano classification [24]. Written informed consent was obtained from each patient before ASCT. This study was approved by the local institutional review board.

### Statistical analysis

Overall survival (OS) was defined as time from the day of ASCT until death from any cause, with censoring of patients known to be alive at the time of last follow-up. PFS was calculated from the day of ASCT until relapse or death. Both OS and PFS were estimated using the Kaplan–Meier method. The log-rank test was used for univariate analysis. Non-relapse mortality (NRM) was defined as death without relapse or progression of disease and when given as a percentage defines the patient sample observed as reference for the denominator. Probabilities of NRM and relapse were calculated using cumulative incidence estimates to accommodate competing risks. The Cox proportional hazard regression model was used for multivariate analysis with backward selection of risk factors. *p* values < 0.05 were defined as significant. Statistical analyses were performed using IBM SPSS statistics, version 26.0 (IBM Corp., Armonk, NY) and R software package, version 3.6.1 (R Foundation, Vienna, Austria; https://www.r-project.org).

## Results

### Baseline characteristics

A total of 247 patients diagnosed with DLBCL, MCL, HL, and PCNSL underwent ASCT between 2002 and 2019 and met the inclusion criteria. Thirty-two percent (*n* = 80) of these patients were diagnosed with DLBCL including 6% (*n* = 16) with transformed indolent lymphoma, 30% (*n* = 75) of patients with MCL, 19% (*n* = 47) with HL, and 18% (*n* = 45) with PCNSL. All HL patients were diagnosed with classical Hodgkin lymphoma. Detailed patient characteristics are summarized in Table 1. The median follow-up time of surviving patients was 36 months (range 0–163 months). The median age at ASCT of all patients was 60 years (range 19–78 years). Patients with HL were of younger age, with a median of 40 years at ASCT. Forty-seven percent of the patients received ASCT consolidation as part of the first-line treatment (predominantly patients with MCL or PCNSL), whereas 53% of the patients received ASCT for relapsed or primary refractory disease after at least two lines of prior therapy. Ten percent of the patients received ASCT after third-line therapy or beyond. Sixty-two percent of patients presented with extranodal involvement at diagnosis and 57% had stage III/IV disease. The conditioning regimens prior to ASCT consisted of carmustine/BCNU, etoposide, cytarabine, and melphalan with or without rituximab ([R]-BEAM) in 73% (*n* = 180) of the cases, whereas 21% (*n* = 52) received thiotepa-based regimens, 4% (*n* = 11) underwent total body irradiation (TBI)-based regimens, and 2% (*n* = 4) were treated with other regimens such as cytarabine and melphalan or high-dose BCNU. Lymphoma-specific information on the conditioning regimens is summarized in Table 1. Remission status prior to ASCT was available for nearly all patients (95% of patients), of which 34% were noted to be in CR and 61% in PR. The median time from start of salvage treatment prior to ASCT was 4 months (range 1–8 months). The median number of infused stem cells was 4.9 × 10^6^ CD34^ +^ cells per kilogram body weight (range 1.2–25 × 10^6^).

**Table 1:** Patient characteristics

	DLBCL	MCL	HL	PCNSL	Total
No. of patients	80	75	47	45	247
Age at diagnosis (years)					
Median	61	60	31	60	58
Range	31–78	42–74	16–71	20–74	16–78
Age at ASCT (years)					
Median	63	61	40	60	60
Range	32–78	42–75	19–72	20–74	19–78
Sex [no. (%)]					
Male	50 (62)	62 (83)	31 (66)	19 (42)	162 (66)
Female	30 (38)	13 (17)	16 (34)	26 (58)	85 (34)
Ann-Arbor stage [no. (%)]					
III/IV	52 (65)	69 (92)	21 (45)	0	142 (57)
B-symptoms [no. (%)]	23 (29)	25 (33)	20 (43)	2 (4)	69 (28)
Extranodal involvement at diagnosis [no. (%)]	48 (60)	42 (56)	17 (36)	45 (100)	153 (62)
Lines of therapy before ASCT [no. (%)]					
1	15 (19)	66 (88)	1 (2)	35 (78)	117 (47)
2	55 (69)	7 (9)	35 (74)	9 (20)	106 (43)
≥ 3	10 (12)	2 (3)	11 (23)	1 (2)	24 (10)
ASCT conditioning regimen [no. (%)]					
(R-)BEAM or similar	71 (89)	62 (83)	47 (100)	0	180 (73)^1^
Thiotepa-based	8 (10)	0	0	45 (100)	52 (21)
TBI-based	0	11 (15)	0	0	11 (4)
Others	1 (1)	2 (3)	0	0	4 (2)
Remission status at ASCT [no. (%)]					
Complete remission	32 (40)	33 (44)	5 (11)	15 (33)	85 (34)
Partial remission	48 (60)	42 (56)	32 (68)	28 (62)	150 (61)
Unknown	0	0	10 (21)	2 (4)	12 (5)
Number of reinfused CD34 + stem cells (10^6^/kg)					
Median	4.6	4.4	5.7	5.3	4.9
Range	1.8–20.7	1.9–15.7	1.9–14.0	1.2–25.0	1.2–25.0
Interval from diagnosis to ASCT (months)					
Median	15	6	33	5	8
Range	2–172	4–133	2–338	2–40	2–338
Interval from start of salvage treatment to ASCT (months)					
Median	3	5	2	4	4
Range	1–6	1–8	1–5	1–6	1–8
Survivor follow-up (months)					
Median	26	34	52	25	36
Range	1–111	0–140	1–163	1–97	0–163

^1^Dose reduction in 7 (3%) patients, mainly due to impaired lung function (4 of these patients were ≥ 65 years)

*DLBCL* diffuse large B-cell lymphoma, *MCL* mantle cell lymphoma, *HL* Hodgkin lymphoma, *PCNSL* primary CNS lymphoma, *ASCT* autologous stem cell transplantation, *(R-)BEAM* (rituximab) carmustine/BCNU etoposide cytarabine melphalan

### Outcome and toxicity

Key outcome parameters and the toxicity profile of our cohort are depicted in Table 2. Leukocyte and thrombocyte engraftments were documented at a median of 10 days (range 7–35 days) and 13 days (range 7–42 days) after ASCT, respectively. Non-hematologic adverse events grade 3 or higher after the common terminology criteria for adverse events (CTCAE) occurred in 83% of the patients (infections 66%, mucositis 44%). The cumulative incidence of deaths in remission until day 100 after ASCT was 4% (10 patients), with infections representing the most common cause of death. Complete remission as best response after ASCT was confirmed in 80% of the patients. Eleven percent of all patients achieved a PR, whereas 3% had progressive disease (PD) at first remission assessment. The cumulative incidences of relapse at 1, 3, and 5 years were 15.8% (95% CI: 11.2–20.4%), 23.5% (95% CI: 18.1–28.9%), and 25.9% (95% CI: 20.3–31.5%), respectively. Among the different lymphoma entities, we noted a cumulative incidence of relapse at 3 years in 21.3% (95% CI: 9.2–33.4%) of HL patients, 20.0% (95% CI: 7.9–32.1%) of PCNSL patients, 26.0% (95% CI: 16.0–36.0%) of DLBCL patients, and 22.7% (95% CI: 12.9–32.4%) of MCL patients. In the entire cohort, 16% (*n* = 31) of the patients underwent subsequent allogeneic stem cell transplantation (alloSCT) either shortly after ASCT (*n* = 6) or after a subsequent relapse or progression (*n* = 20). Five additional patients received alloSCT for a disease other than the indication for ASCT: secondary myelodysplastic syndrome (MDS) (*n* = 2), acute myeloid leukemia (*n* = 1), and other lymphomas (*n* = 2). Thirty-seven percent (28 of 75 patients) of patients with MCL received rituximab maintenance treatment after ASCT. Except for one of these patients, all other received rituximab maintenance after ASCT as part of the first-line therapy. In the HL cohort, 2 patients with PR at ASCT received brentuximab vedotin maintenance after ASCT and did not develop relapse.

**Table 2:** Outcome and toxicities

	DLBCL	MCL	HL	PCNSL	Total
No. of patients	80	75	47	45	247
Leukocyte engraftment (day after ASCT)					
Median	10	10	10	10	10
Range	8–21	8–24	7–13	8–35	7–35
Platelet engraftment (day after ASCT)					
Median	13	13	12	11	13
Range	7–42	8–29	7–16	7–25	7–42
Adverse events grade ≥ 3 CTCAE					
Nausea	4 (5)	2 (3)	0	4 (9)	10 (4)
Mucositis	31 (39)	26 (35)	10 (27)	20 (44)	87 (35)
Infection	60 (75)	57 (76)	37 (79)	37 (82)	191 (77)
Renal toxicity	1 (1)	0	0	1 (2)	2 (1)
Liver toxicity	1 (1)	1 (1)	0	0	2 (1)
Others^1^	2 (3)	2 (3)	3 (6)	3 (7)	10 (4)
Remission status after ASCT [no. (%)]					
Complete remission^3^	60 (75)	68 (91)	34 (72)	35 (78)	197 (80)
Partial remission	12 (15)	4 (5)	6 (13)	6 (13)	28 (11)
Progressive disease	3 (4)	1 (1)	0	3 (7)	7 (3)
Unknown	5 (6)	2 (3)	7 (15)	1 (2)	15 (6)
Relapse [no. (%)]	22 (28)	24 (32)	11 (23)	11 (24)	68 (28)
Follow-up allogeneic SCT [no. (%)]	10 (13)	7 (9)	14 (30)	0	31 (16)
Death [no. (%)]	35 (44)	20 (27)	8 (17)	12 (27)	75 (30)
Reasons of death [no. (% of deaths)]					
Lymphoma-related	22 (63)	12 (60)	5 (62)	9 (75)	48 (64)
Infection	6 (17)	7 (35)	3 (38)	1 (8)	17 (23)
Cardiac event	2 (6)	0	0	1 (8)	3 (4)
Second neoplasia	2 (6)	0	0	1 (8)	3 (4)
Others^2^	2 (6)	1 (5)	0	0	3 (4)
Not known	1 (3)	0	0	0	1 (1)

^1^Anaphylaxis, ARDS, atrial fibrillation, atrial flutter, graft failure, neurologic deficit, seizure, thrombosis

^2^Hypoxia caused by aspiration, cerebral aneurysm, respiratory failure with unclear interstitial lung disease

^3^Including patients with CR pre-ASCT

*DLBCL* diffuse large B-cell lymphoma, *MCL* mantle cell lymphoma, *HL* Hodgkin lymphoma, *PCNSL* primary CNS lymphoma, *ASCT* autologous stem cell transplantation

During the follow-up period, 75 patients died. The leading cause of death was lymphoma-related, accounting for 63% of deaths. The cumulative incidences of NRM at 1, 3, and 5 years were 8.1% (95% CI: 4.6–11.6%), 8.9% (95% CI: 5.3–12.5%), and 9.7% (95% CI: 5.9–13.5%), respectively. Median PFS for all patients was 71 months (95% CI: 52–109 months), while median OS was not reached.

**Fig. 1:**
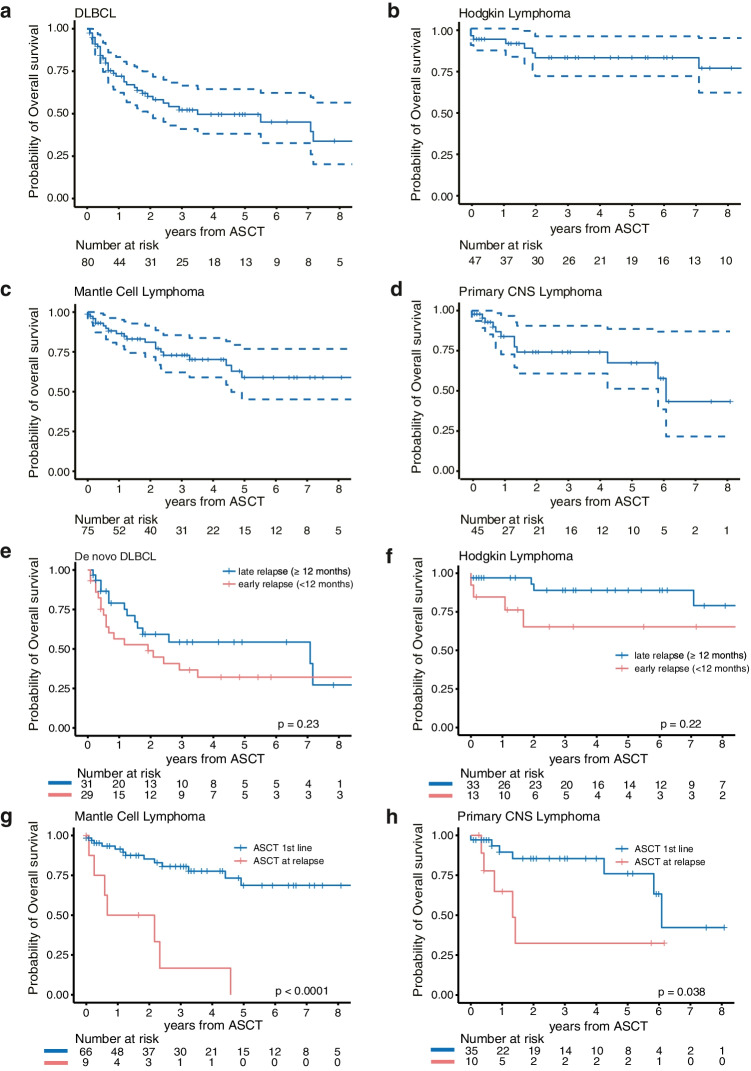
Kaplan–Meier survival estimates showing overall survival (OS) for **a** DLBCL patients, **b** Hodgkin lymphoma patients, **c** mantle cell lymphoma patients, and **d** primary CNS lymphoma patients. OS curves stratified by time of relapse (< 12 months vs. ≥ 12 months) after first-line treatment in the **e** de novo DLBCL and **f** Hodgkin lymphoma subcohorts. Conditional OS by line of therapy of ASCT (first-line [1st line] vs. at relapse) for the **g** mantle cell lymphoma and **h** primary CNS lymphoma subcohorts

Outcome varied between different lymphoma entities. Mean OS was 55 months (95% CI: 43–66 months) in DLBCL patients, in HL patients 132 months (95% CI: 112–152 months) (Fig. 1a–b). For patients with MCL, mean OS was 94 months (95% CI: 77–111 months) and for PCNSL patients 65 months (95% CI: 51–79 months) (Fig. 1c–d).

**Fig. 2:**
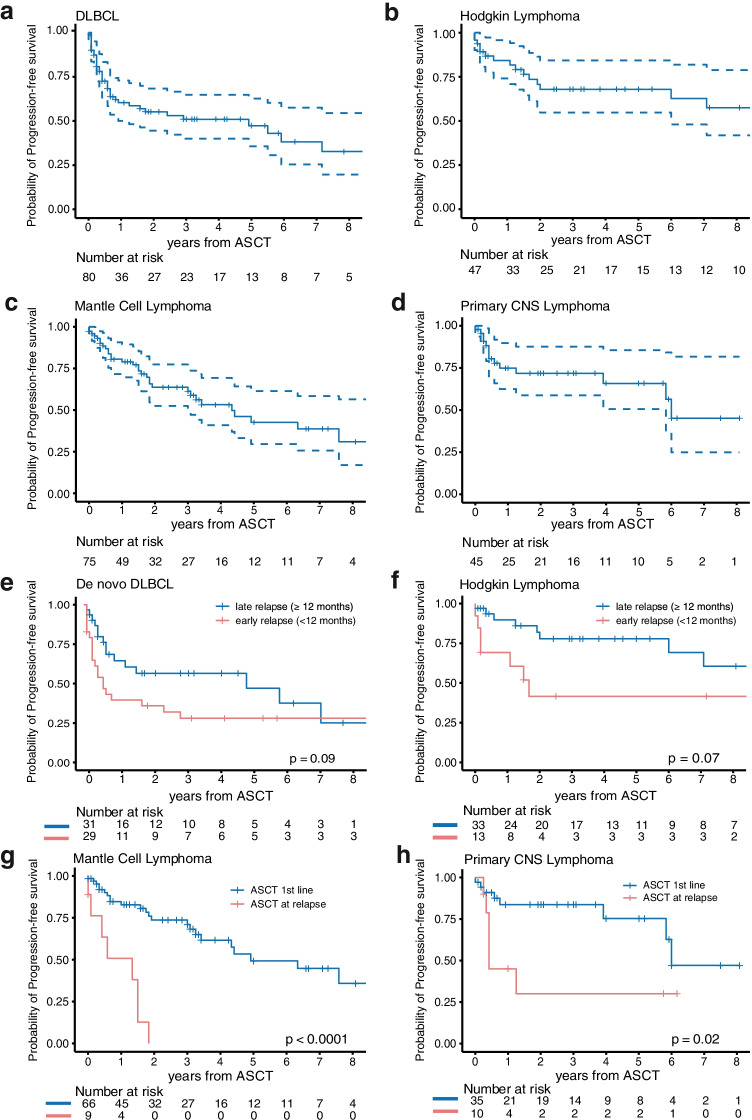
Kaplan–Meier survival estimates showing progression-free (PFS) for **a** DLBCL patients, **b** Hodgkin lymphoma patients, **c** mantle cell lymphoma patients, and **d** primary CNS lymphoma patients. PFS by time of relapse (< 12 months vs. ≥ 12 months) after first-line treatment for patients with **e** de novo DLBCL and **f** for patients with Hodgkin lymphoma. PFS by line of therapy of ASCT (first-line [1st line] vs. at relapse) for **g** patients with mantle cell lymphoma and **h** patients with primary CNS lymphoma subcohorts

Kaplan–Meier estimates for PFS demonstrated similar trends. Mean PFS in DLBCL patients was 51 months (95% CI: 40–62 months), and 97 months (95% CI: 74–120 months) in HL patients (Fig. 2a–b). For the MCL subcohort, mean PFS was 65 months (95% CI: 49–82 months), and for the PCNSL subcohort 63 months (95% CI 49–77 months) (Fig. 2c–d).

De novo DLBCL patients experiencing an early relapse (< 12 months) after first-line treatment tended to have inferior PFS (*p* = 0.09), compared to those with late relapse, with a 2-year PFS of 35.9% (95% CI: 21.9–59.0%) vs. 56.5% (95% CI: 40.5–78.8%), respectively (Fig. 1e, Fig. 2e). For the respective OS, no significant differences were noted. In HL patients, we observed a similar trend towards inferior PFS (*p* = 0.07) of 41.5% (95% CI: 20.7–83.3%) vs. 77.8% (95% CI: 63.5–95.4%) at 2 years for patients encountering an early relapse, while OS remained not significantly influenced (*p* = 0.22) (Fig. 1f, Fig. 2f).

For MCL and PCNSL, the majority of patients received ASCT as consolidation in first line, while approximately 16% of these patients underwent ASCT as a part of salvage treatment mostly due to higher patient age, year of diagnosis, participation in clinical trials, and response to first-line treatment. ASCT in first line for MCL and PCNSL showed significantly improved PFS and OS compared to those treated with ASCT at relapse (Fig. 1g–h, Fig. 2g–h).

### Prognostic factors for outcome after ASCT

**Fig. 3:**
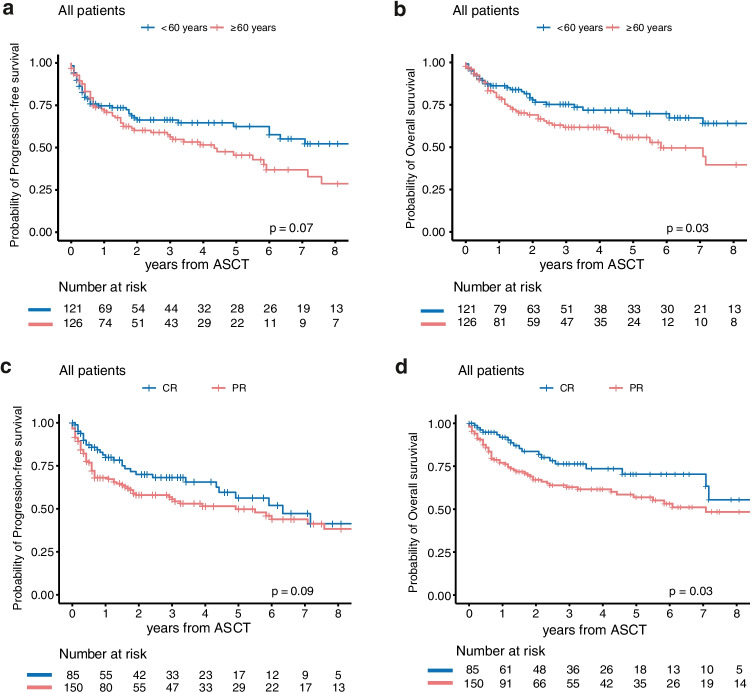
Kaplan–Meier survival estimates showing progression-free survival and overall survival stratified by **a**–**b** age at ASCT and **c**–**d** remission status at ASCT

As shown in Table 3 and Fig. 3, a variety of risk factors showed prognostic impact on PFS and OS at 3 years after ASCT in univariate analysis. Variables significantly associated with superior OS after ASCT were CR as remission status before ASCT, age < 60 years at ASCT, and the amount of infused CD34^+^ cells > 4.9 Mio./kg. Specifically, for those patients who received ASCT with ≤ 4.9 Mio./kg, higher NRM rates were observed when compared to those who received > 4.9 Mio./kg (*p* = 0.025). However, we did not observe significant differences in the amount of harvested CD34^+^ cells throughout the entire study period, leukocyte engraftment or specific infections such as CMV reactivations between both CD34 groups (data not shown). In the multivariate analysis, remission status before ASCT, age at ASCT as continuous variable, and the amount of CD34^+^ cells (≤ 4.9 Mio. CD34^+^ cells/kg versus > 4.9 Mio. CD34^+^ cells/kg) could be identified as independent risk factors for both OS (age: HR 1.04, 95% CI 1.02–1.06, *p* < 0.01; remission status: HR 0.45, 95% CI 0.26–0.78, *p* < 0.01; > 4.9 Mio./kg of infused cells: HR 1.88, 95% CI 1.16–3.05, *p* = 0.01) and PFS (age: HR 1.02, 95% CI 1.00–1.04, *p* = 0.02; remission status: HR 0.62, 95% CI 0.39–0.97, *p* = 0.04; number of infused cells: HR 1.70, 95% CI 1.12–2.57, *p* = 0.01).

**Table 3:** Univariate analysis

	*n* (% of patients)	3y-PFS (95% CI)	*p* value	3y-OS (95% CI)	*p* value
Age at ASCT					
< 60 years	121 (49)	66 (61–71)	0.07	75 (71–80)	0.03
≥ 60 years	126 (51)	56 (51–61)		62 (57–67)	
Gender					
Male	85 (34)	63 (58–67)	0.69	60 (54–66)	0.20
Female	162 (66)	58 (52–64)		72 (68–76)	
Ann-Arbor stage					
I/II	102 (42)	65 (60–70)	0.12	73 (68–78)	0.33
III/IV	142 (58)	58 (54–62)		65 (60–69)	
B-symptoms					
Yes	70 (37)	56 (50–62)	0.11	67 (60–73)	0.54
No	117 (63)	63 (59–67)		69 (65–73)	
Extranodal involvement					
Yes	152 (62)	57 (53–61)	0.09	64 (59–68)	0.14
No	95 (38)	67 (62–72)		75 (70–80)	
Remission status before ASCT					
CR	85 (36)	68 (62–74)	0.09	76 (71–82)	0.03
PR	150 (64)	56 (51–61)		63 (58–67)	
Conditioning regimen			0.48		0.83
(R-)BEAM	180 (73)	58 (54–62)		67 (63–71)	
Thiotepa-based	52 (21)	71 (64–78)	0.26	75 (68–82)	0.67
TBI-based	11 (4)	55 (40–70)	0.75	55 (40–70)	0.62
Others	4 (2)	75 (53–97)	0.31	75 (53–97)	0.53
Number of infused cells					
≤ 4.9 Mio./kg	116 (50)	52 (47–58)	0.01	56 (50–61)	0.01
> 4.9 Mio./kg	118 (50)	67 (62–72)		79 (75–83)	

*ASCT* autologous stem cell transplantation, *(R-)BEAM* (rituximab) carmustine/BCNU etoposide cytarabine melphalan, *TBI* total body irradiation, *n* number of patients, *3y-PFS* 3-year progression-free survival, *CI* confidence interval, *3y-OS* 3-year overall survival

## Discussion

In this retrospective study, we analyzed the characteristics and outcome of patients treated with high-dose chemotherapy followed by ASCT in different entities of aggressive B-cell lymphoma. Our data sustain the existing evidence that HD chemotherapy followed by ASCT as part of salvage treatment strategies for relapsed diseases or as part of the first-line treatment in MCL or PCNSL is effective and is capable to allow cure or at least long-term disease control in a substantial number of patients.

In our study, remission status prior to ASCT was identified as important independent risk factor for survival outcome. Those patients undergoing ASCT with a remission status other than complete remission showed an inferior survival in our cohort. This observation is in line with several previously reported studies, in particular for HL and DLBCL patients, suggesting that ASCT might be more effective in patients with chemosensitive disease [25–27]. For patients with chemo-refractory disease, other treatment options such as CAR T cell therapies, antibody-based therapies, targeted treatments with tyrosine kinase inhibitors, or allogeneic stem cell transplantation should be considered depending on the specific lymphoma entity, molecular characteristics, and comorbidities.

Secondly, multivariate analysis identified age as continuous parameter as a prognostic factor for OS and PFS after ASCT. However, in univariate analysis, a cutoff of 60 years and older, as applied in the IPI score, did not show significant differences in outcomes. While previous retrospective studies showed inconsistent results on the prognostic value of age, our data demonstrate that ASCT in elderly patients is feasible unless no significant comorbidities exist [25–27]. Furthermore, the amount of infused CD34^+^ cells was identified as a potential prognostic factor, with higher amounts of infused CD34^+^ cells being associated with favorable outcome. This finding is in line with a Swedish study that previously reported a similar trend with regard to the infused number of CD34^+^ cells [28]. In our cohort, we observed a higher NRM rate for patients who received lower numbers of transfused stem cells, suggesting a potentially higher risk for infectious complications. However, time to leukocyte engraftment did not differ significantly between patients with low or high number of transfused stem cells and patients receiving lower numbers of transfused stem cells were not those with more intense pre-treatment prior ASCT. Hence, the number of infused stem cells might be a surrogate parameter, e.g., for impaired immune cell recovery resulting in a higher susceptibility to severe infection. Consequently, any further conclusion with regard to treatment decision based on the available stem cell graft is not possible.

While most lymphoma relapses (81%) occurred within 24 months after ASCT, 19% of relapses were documented as late events beyond 2 years after ASCT. Lymphoma relapse was the most frequent reason of death accounting for 64% of all deaths observed in our study. These results underline that disease relapse remains a leading cause even in the late post-ASCT phase [29–31].

The most frequently observed adverse events comprised febrile neutropenia, mucositis, and nausea, as reported before elsewhere [10, 15–18, 32–35]. The observed NRM of 4% at day 100 after ASCT was in a similar range as compared to other recent retrospective studies [21, 22, 26, 28]. Although NRM mostly occurred within 3 years after ASCT, NRM steadily increases over time, with infections representing the leading cause of late NRM events. Thus, extended surveillance for patients after ASCT particularly monitoring infections has to be considered.

Of note, DLBCL patients in our cohort achieved a 3-year PFS of 51% after transplantation, comparable to the results of the CORAL study [8]. Patients with an early relapse after diagnosis (≤ 12 months) of DLBCL showed a trend towards inferior outcome after ASCT when compared to late relapses, which is also in line with published data [11, 8]. Most likely, our observation did not reach statistical significance due to the limited number of patients in this subcohort. For patients with an early relapse, chemo-refractory disease or relapse after ASCT, both, CAR T cell therapy and an allogeneic stem cell transplantation, might be more effective treatment options [36–41]. For patients who are ineligible for ASCT or with relapse after ASCT, CAR T cell therapy with tisagenlecleucel demonstrated an overall response rate of 52%. The relapse-free survival estimate 12 months after initial response was 65% [37]. Allogeneic stem cell transplantation might be an additional treatment alternative for selected patients. In a study by Glass et al., PFS at 1 year after alloSCT was 45% [42]. However, results from prospective trials evaluating ASCT in comparison to CAR T cell approaches are still awaited. These studies bear the potential to define new standards in the future.

Although patients with HL generally have an excellent prognosis, treatment of HL relapse remains challenging. In relapsed or refractory states of disease, ASCT is known to induce sustained remissions in more than half of these patients [9, 10, 12], which is similarly reflected by the present study. For those patients ineligible for HD chemotherapy with ASCT or with relapse thereafter, less-toxic approved treatment approaches such as PD-L1 blockade (e.g., nivolumab or pembrolizumab) and other targeted therapy approaches (e.g., brentuximab vedotin) are reasonable alternatives [43–45]. For example, treatment with brentuximab vedotin in relapsed or refractory HL patients resulted in an estimated 5-year OS and PFS of 41% and 22%, respectively [43]. First results of the ongoing randomized phase III KEYNOTE-204 study comparing pembrolizumab to brentuximab vedotin for relapsed or refractory HL patients after ASCT demonstrated a superior PFS for pembrolizumab [46].

For MCL and PCNSL, current guideline recommendations suggest ASCT as part of first-line treatment protocols. The outcome of MCL and PCNSL patients in the present study is well in line with reported data from larger trials in both entities [17, 18, 32–34, 47]. In patients with MCL, effectiveness of CAR T cell therapy in relapsed or refractory disease has been shown in a recent phase 2 trial, demonstrating durable remissions in the majority of patients. The estimated PFS at 12 months was 61% [48].

In conclusion, our data verify current treatment recommendations with regard to HD chemotherapy followed by ASCT for the treatment of aggressive B-cell lymphomas.
